# Supplementary material for: Stability Oracle: a structure-based graph-transformer framework for identifying stabilizing mutations
Source: Nat Commun. 2024 Jul 23;15:6170. doi: 10.1038/s41467-024-49780-2 (PMC11266546; doi:10.1038/s41467-024-49780-2)
Supplement: Supplementary file 1 — Supplementary Information [file 41467_2024_49780_MOESM1_ESM.pdf]

A Supplementary Data

A.1 Running time on different proteins.

PDB ID	# Residues	Per-Residue Graph Generation Time (s)	Per-Residue Inference Time (s)
4DCH	217	0.0193±0.0001	0.0783±0.0042
4AJY	355	0.0108±0.0001	0.0609±0.0012
2R7E	1337	0.0273±0.0001	0.0457±0.0001
6VSB	2905	0.0514±0.0001	0.0422±0.0004

Supplementary Table 1. We present the time to generate the masked microenvironment graph per residue (data processing time) and predict the $\Delta\Delta G$ for the 20 amino acids per residue (inference time). These performance benchmarks are averages across 3 runs and were conducted with one core of a 2.00 GHz Intel(R) Xeon(R) Gold 6338 CPU for graph generation and one A100 GPU for model inference.

A.2 Evaluating our structural amino acid embeddings v.s. one-hot encodings.

Representation	Test Set	*Pearson*	*Spearman*	*RMSE*	*AUROC*	Precision	Recall	Accuracy
Structural AA Embeddings	T2837	0.59	0.62	1.65	0.81	0.46	0.55	0.82
One-Hot Encoding	T2837	0.48	0.44	1.90	0.68	0.35	0.44	0.66

Supplementary Table 2. We present the comparison of two different amino acid representations on the T2837 test set: Structural amino acid embeddings, and one-hot encoding. The structural amino acid embeddings are 128-dim vectors obtained from the weights of the final layer in the self-supervised pre-trained model. The table shows the average values of the Pearson correlation coefficient, Spearman correlation coefficient, Root Mean Squared Error (RMSE), Area Under the Receiver Operating Characteristic Curve (AUROC), Precision, Recall, and Accuracy. Results are the average of three independently trained models on the cDNA117K dataset.

A.3 Evaluating different Data Augmentation Techniques

Augmentation	Test Set	*Pearson*	*Spearman*	*RMSE*	*AUROC*	Precision	Recall	Accuracy
No	T2837	0.55	0.55	1.71	0.78	0.32	0.53	0.78
TR	T2837	0.58	0.59	1.68	0.80	0.44	0.49	0.80
TP	T2837	0.59	0.62	1.64	0.81	0.47	0.53	0.82

Supplementary Table 3. We present the comparison of different augmentation techniques. The table shows the average values of the Pearson correlation coefficient, Spearman correlation coefficient, Root Mean Squared Error (RMSE), Area Under the Receiver Operating Characteristic Curve (AUROC), Precision, Recall, and Accuracy. Results are the average of three independently trained models on the cDNA117K dataset.

T2837 TP-Only AlphaFold Structure	*Pearson*	*Spearman*	*RMSE*	*AUROC*	Precision	Recall	Accuracy
WT	0.67	0.66	1.51	0.81	0.67	0.66	0.76
FROM	0.65	0.62	1.56	0.79	0.63	0.64	0.74
TO	0.64	0.61	1.58	0.79	0.60	0.63	0.74

Supplementary Table 4. Stability Oracle's T2837 TP-only performance on different AlphaFold structure datasets. To evaluate the impact the local structure has on generalization, we created three AlphaFold structural datasets of the TP mutations of T2837 (7720 mutations) where we vary the amino acid at the mutation position: WT, FROM, TO. Specifically, The WT, FROM, and TO dataset has the wildtype, "FromAA", and "ToAA" amino acid present at the mutation position, respectively.

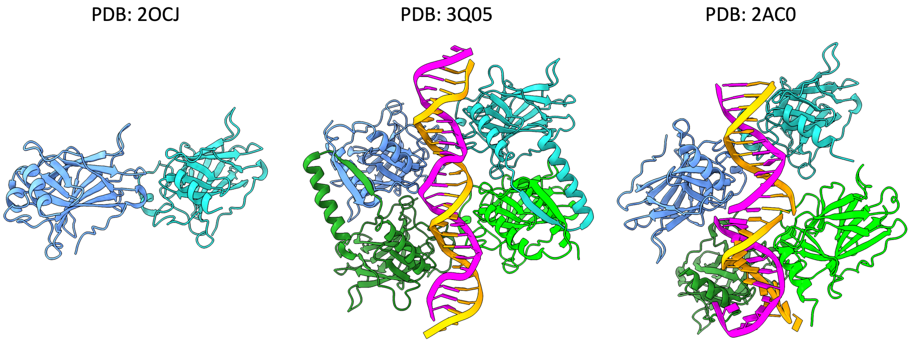

PDB code	Resolution	biological assembly	Position	*Pearson*	*Spearman*	*RMSE*	*AUROC*	Precision	Recall	Accuracy
2OCJ	2.05 Å	homodimer	94-312	0.73	0.68	1.50	0.80	0.46	0.91	0.73
3Q05	2.40 Å	homotetramer complexed with a DNA helix	94-326	0.75	0.80	1.48	0.83	0.55	0.91	0.81
2AC0	1.80 Å	homotetramer complexed with two DNA helices	94-293	0.78	0.79	1.70	0.86	0.64	0.84	0.83
-	-	-	-	0.75±0.02	0.76±0.05	1.56±0.10	0.83±0.02	0.55±0.07	0.89±0.03	0.79±0.04

Supplementary Figure 1. Case study of Stability Oracle's performance on different P53 structures (PDBs: 2OCJ, 3Q05, 2AC0). This experiment demonstrates the sensitivity of Stability Oracle to the quality of the input structure.

A.4 Thermodynamic Data Augmentation Techniques: Reversibility vs Permutations

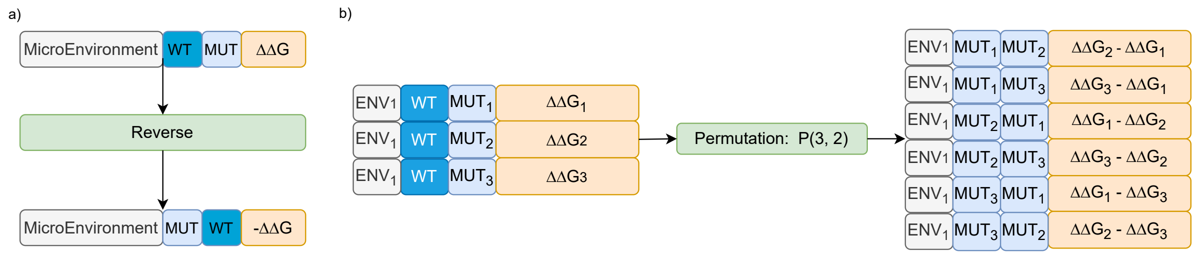

Supplementary Figure 2. Thermodynamic data augmentation techniques applied to our datasets. a) Diagram describing how Thermodynamic Reversibility works (TR) b) Diagram describing how Thermodynamic Permutations works (TP). In this example, we expand 3 experimental $\Delta\Delta G$ instances into an additional 6 $\Delta\Delta G$ instances. Note: TP augmented data instances do not include the wildtype amino acid.

A.5 Distribution of experimental datasets by mutation type with Thermodynamic Reversibility.

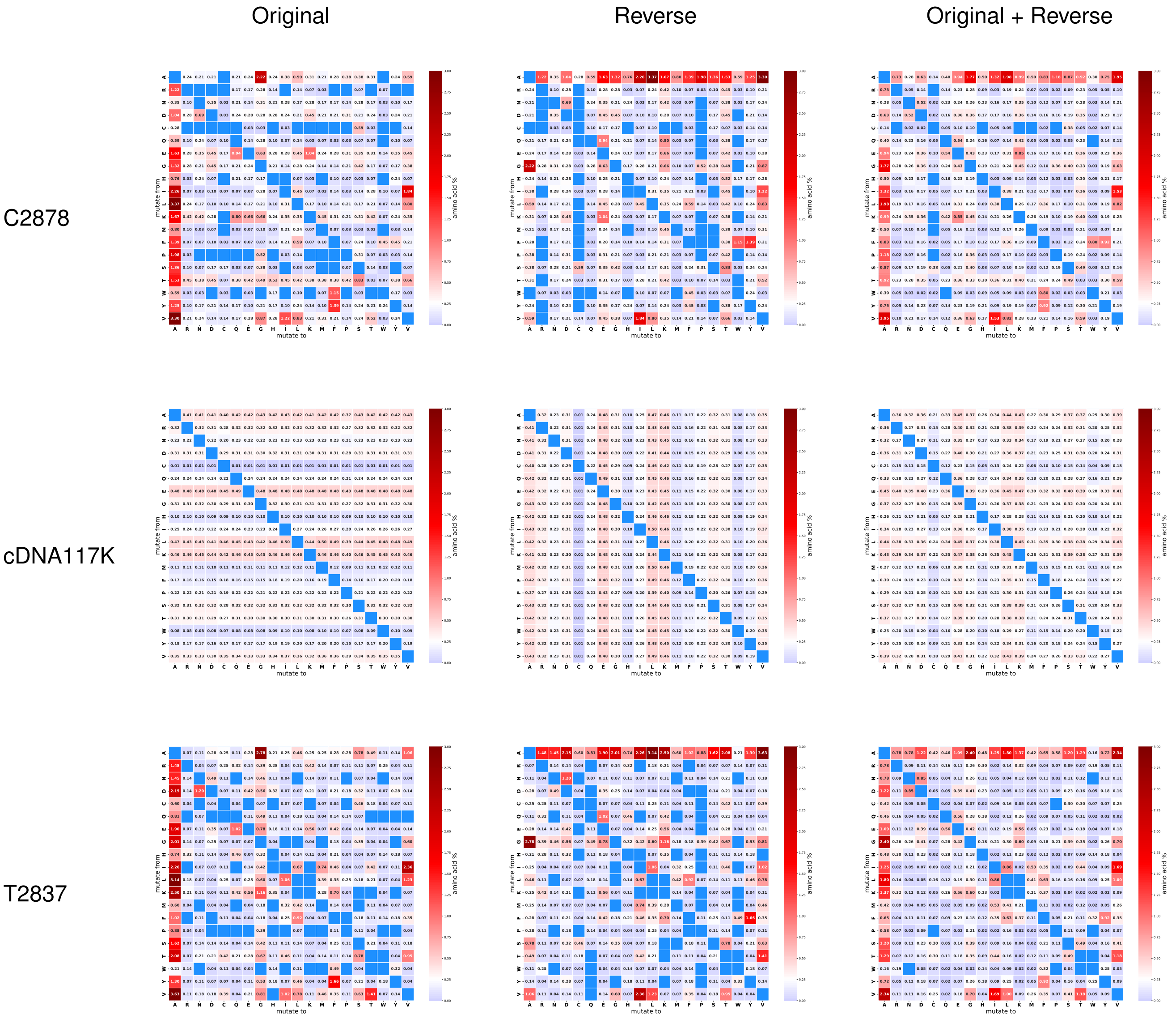

Supplementary Figure 3. Heatmap of C2878, cDNA117K, and T2837. The first column shows the mutation type distribution of the original data, the middle column shows the TR augmented data, and the right column shows the original + TR datasets. For C2878 + TR, the missing mutation types are R->C, C->H, C->K, C->M, C->P, Q->W, C->R, H->C, K->C, M->C, P->C, W->Q. For T2837 + TR, the missing mutation types are: W->N, P->C, W->P, W->T, T->W, P->W, C->P, N->W, K->L, L->K.

A.6 Distribution of experimental datasets by mutation type with Thermodynamic Permutations.

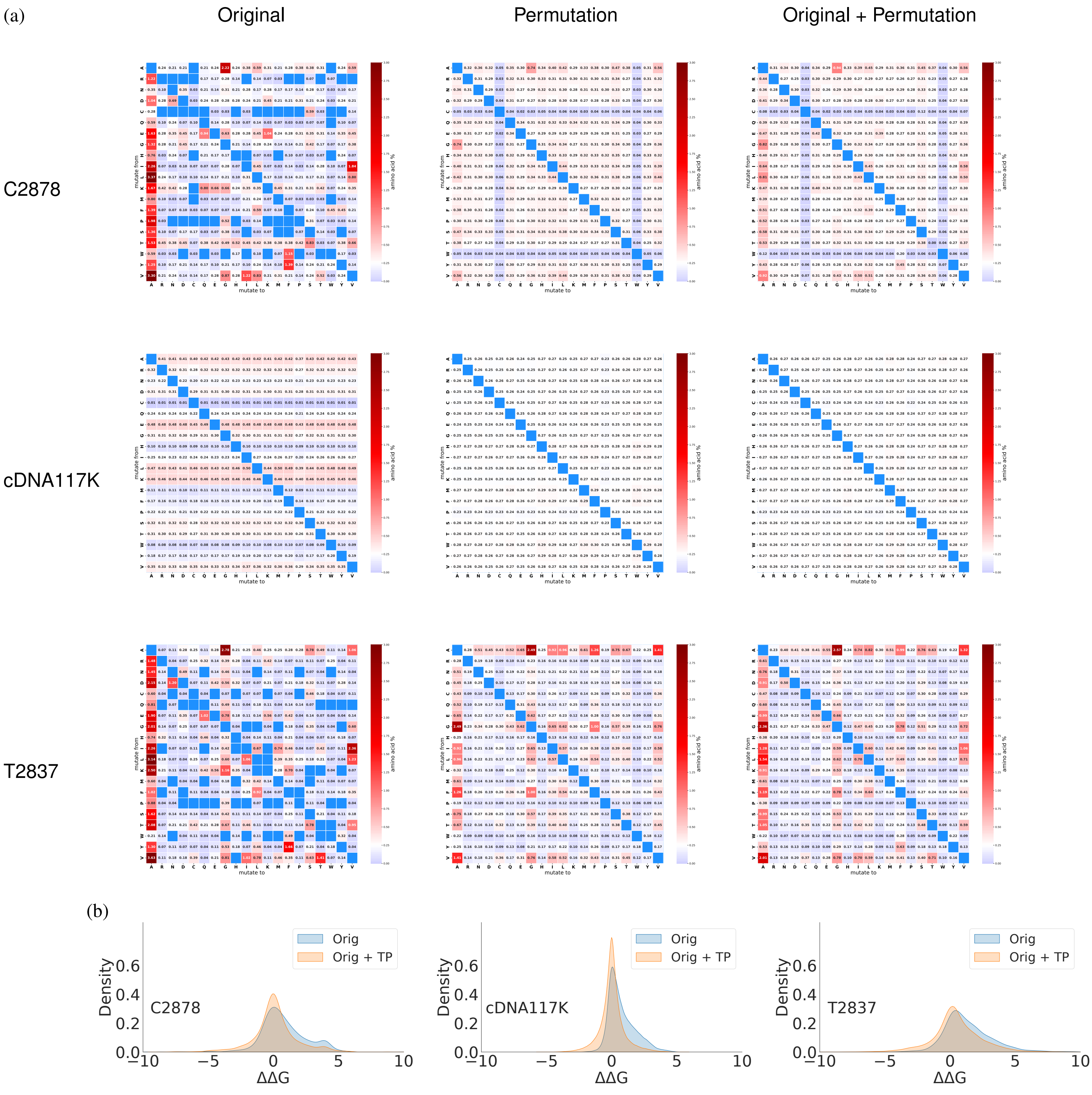

Supplementary Figure 4. (a) Heatmap of the mutation type distribution of C2878, cDNA117K, and T2837. The first column shows the original data, the middle column shows the TP augmented data, and the right column shows the original + TP datasets. (b) Density plots that superimpose the original datasets and the TP augmented dataset. This plot highlights how TP augmentation adds stabilizing mutations that do not include the wildtype amino acids.

A.7 Stabilization ratio for the 380 mutation types.

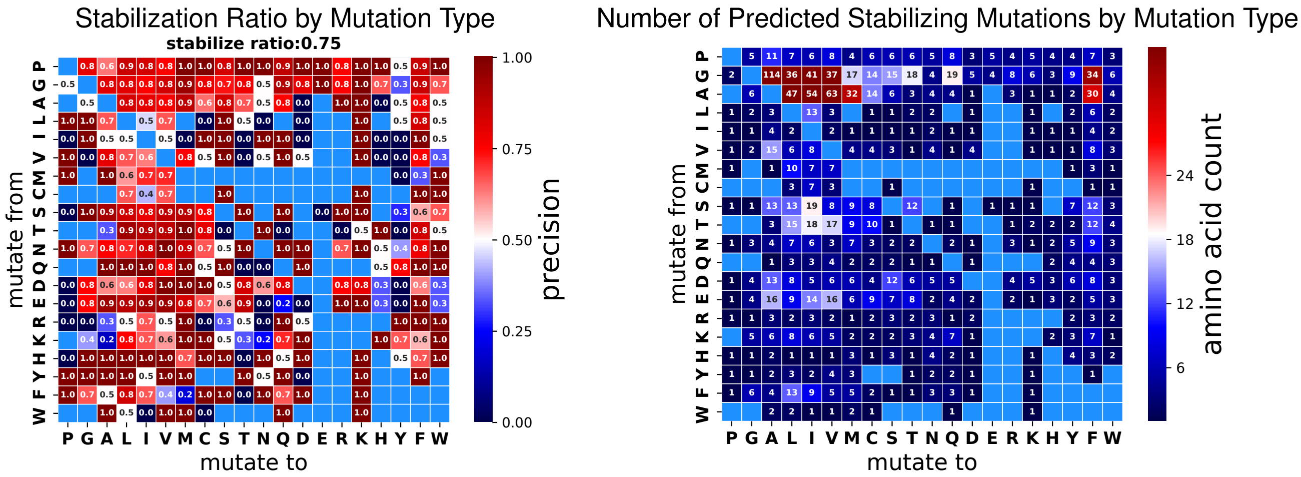

Supplementary Figure 5. Distribution of Stability Oracle's stabilizing predictions with a stability threshold of $\Delta\Delta G < -0.5$ kcal/mol on T2837 + TP. Left shows the ratio of predictions that are experimentally stabilizing for each mutation type. Right shows the number of mutations predicted to be stabilizing for each mutation type, highlighting the lack of data for evaluation.

A.8 Performance of Stability Oracle and Prostata-IFML on Common Test Sets

Test Set	*Pearson*	*Spearman*	*RMSE*	*AUROC*	Precision	Recall	Accuracy
S-sym	0.72	0.70	1.22	0.87	0.69	0.52	0.83
S-sym Reverse	0.72	0.70	1.19	0.87	0.82	0.95	0.80
Myoglobin	0.68	0.66	0.90	0.82	0.56	0.36	0.74
Myoglobin Reverse	0.68	0.66	0.89	0.81	0.77	0.94	0.74
S669	0.52	0.53	1.43	0.75	0.58	0.37	0.75
S669 Reverse	0.52	0.53	1.43	0.75	0.80	0.92	0.75
P53	0.73	0.68	1.50	0.80	0.46	0.91	0.73
P53 Reverse	0.73	0.68	1.51	0.80	0.75	0.95	0.75

(a)

Test Set	*Pearson*	*Spearman*	*RMSE*	*AUROC*	Precision	Recall	Accuracy
S-sym	0.55	0.51	1.60	0.75	0.46	0.55	0.72
S-sym Reverse	0.55	0.50	1.61	0.75	0.84	0.78	0.72
Myoglobin	0.54	0.45	0.99	0.67	0.35	0.23	0.67
Myoglobin Reverse	0.54	0.45	0.99	0.69	0.73	0.81	0.65
S669	0.49	0.52	1.61	0.76	0.49	0.41	0.74
S669 Reverse	0.49	0.52	1.61	0.76	0.82	0.83	0.73
P53	0.72	0.70	2.02	0.84	0.47	0.64	0.71
P53 Reverse	0.73	0.71	2.03	0.85	0.87	0.70	0.70

(b)

Supplementary Table 5. In (a), we present the performance of the Stability Oracle on various literature test sets. In (b), we present the performance of Prostata-IFML on various literature test sets. We report the Pearson and Spearman correlation coefficients, root mean square error (RMSE), the area under the receiver operating characteristic (AUROC) curve, Precision, Recall, and Accuracy.

Dataset	# Mutations	Metric	Stability Oracle	Stability Oracle*	Prostata-IFML	Prostata-IFML*	RaSP
All	935	Pearson	0.75	0.71	0.78	0.66	0.72
		Spearman	0.71	0.66	0.79	0.64	0.64
		AUROC	0.84	0.82	0.90	0.82	0.66
		RMSE	1.68	1.69	0.95	1.16	1.42
Quantitative	830	Pearson	0.68	0.64	0.75	0.58	0.60
		Spearman	0.62	0.59	0.76	0.58	0.53
		AUROC	0.81	0.80	0.89	0.80	0.65
		RMSE	1.51	1.54	0.76	0.82	1.33

Supplementary Table 6. Regression and Classification performance of Stability Oracle, Prostata-IFML and RaSP on Gβ1 (PDB:1PGA). * indicates that we retrained on the cDNA117K dataset with PDB:1YU5,1GJS,5UBS,5UCE removed in order to address data leakage (>30% sequence similarity) with Gβ1. We report results on all 935 mutations (qualitative + quantitative) and the 835 mutations quantitative subset as reported in[1]. RaSP was not retrained to address any potential data leakage.

Test Set	Proteins leaked	Proteins total	Mut leaked	Mut total	*Pearson*	*Spearman*	*RMSE*	*AUROC*	Precision	Recall	Accuracy
S-sym	0	15	0	342	0.51	0.47	1.40	0.71	0.44	0.43	0.72
S-sym R					0.51	0.46	1.40	0.72	0.80	0.84	0.72
Myo	0	1	0	134	0.48	0.45	1.01	0.70	0.37	0.19	0.68
Myo R					0.47	0.45	1.01	0.70	0.74	0.89	0.69
p53	0	1	0	42	0.59	0.59	1.77	0.78	0.47	0.64	0.71
p53 R					0.59	0.58	1.77	0.78	0.85	0.74	0.71
S669	9	93	123	669	0.49	0.50	1.45	0.73	0.50	0.37	0.75
S669 R					0.48	0.50	1.45	0.73	0.81	0.89	0.75
t2837	15	126	331	2835	0.42	0.45	2.14	0.72	0.36	0.43	0.73
t2837 R					0.42	0.46	2.13	0.72	0.84	0.85	0.75

(a) Trained on Q1744

Test Set	Proteins leaked	Proteins total	Mut leaked	Mut total	*Pearson*	*Spearman*	*RMSE*	*AUROC*	Precision	Recall	Accuracy
S-sym	15	15	342	342	0.98	0.98	0.35	0.99	0.93	0.87	0.94
S-sym R					0.98	0.98	0.35	0.99	0.93	0.99	0.93
Myo	1	1	134	134	0.72	0.77	0.78	0.87	0.76	0.36	0.78
Myo R					0.73	0.78	0.77	0.86	0.78	0.96	0.78
p53	1	1	42	42	0.60	0.62	1.60	0.80	0.54	0.64	0.76
p53 R					0.58	0.61	1.62	0.79	0.86	0.81	0.76
S669	2	93	33	669	0.50	0.52	1.43	0.74	0.55	0.35	0.76
S669 R					0.50	0.52	1.43	0.74	0.80	0.90	0.76
t2837	35	126	2192	2835	0.77	0.77	1.90	0.88	0.62	0.54	0.84
t2837 R					0.78	0.77	1.90	0.88	0.89	0.91	0.83

(b) Trained on Prostata's dataset for s669

Test Set	Proteins leaked	Proteins total	Mut leaked	Mut total	*Pearson*	*Spearman*	*RMSE*	*AUROC*	Precision	Recall	Accuracy
S-sym	2*	15	75	342	0.50	0.45	1.40	0.71	0.47	0.44	0.73
S-sym R					0.50	0.45	1.41	0.73	0.82	0.83	0.73
Myo	1	1	134	134	0.73	0.79	0.76	0.88	0.83	0.56	0.84
Myo R					0.72	0.78	0.77	0.87	0.85	0.97	0.86
p53	1	1	42	42	0.52	0.58	1.73	0.79	0.44	0.64	0.69
p53 R					0.51	0.56	1.75	0.79	0.85	0.74	0.71
S669	93	93	669	669	0.98	0.99	0.38	1.00	0.97	0.89	0.97
S669 R					0.98	0.99	0.37	1.00	0.97	0.99	0.97
t2837	112	126	1384	2835	0.53	0.59	2.08	0.82	0.52	0.46	0.80
t2837 R					0.53	0.59	2.10	0.82	0.90	0.80	0.77

(c) Trained on Prostata's dataset for ssym. (* these proteins have 40% sequence similarity.)

Test Set	Proteins leaked	Proteins total	Mut leaked	Mut total	*Pearson*	*Spearman*	*RMSE*	*AUROC*	Precision	Recall	Accuracy
S-sym	15	15	342	342	0.98	0.98	0.34	0.99	0.95	0.89	0.94
S-sym R					0.98	0.98	0.34	0.99	0.95	0.98	0.94
Myo	1	1	134	134	0.73	0.79	0.77	0.88	0.85	0.47	0.82
Myo R					0.73	0.79	0.76	0.87	0.80	0.97	0.81
p53	1	1	42	42	0.58	0.62	1.66	0.81	0.47	0.64	0.71
p53 R					0.59	0.63	1.64	0.81	0.86	0.77	0.74
S669	93	93	669	669	0.98	0.99	0.34	0.99	0.97	0.89	0.97
S669 R					0.98	0.99	0.35	0.99	0.96	0.99	0.96
t2837	126	126	2835	2835	0.87	0.87	1.82	0.94	0.74	0.72	0.89
t2837 R					0.86	0.87	1.83	0.93	0.91	0.94	0.87

(d) Trained on Prostata's newly introduced dataset

Supplementary Table 7. Prostata results when trained on datasets introduced in their original paper. A test protein is "leaked" if there is a protein in the training set with greater than 30% sequence similarity. A mutation is "leaked" if it belongs to a "leaked" protein. The new training sets introduced in Prostata filter with a 75% sequence similarity.

A.9 Performance on identifying stabilizing mutations

DataSet	Predict $\Delta\Delta G$ < Threshold	#Data	Experimental $\Delta\Delta G > 0.5$ Destable	Experimental $\|\Delta\Delta G\| \leq 0.5$ Neutral	Experimental $\Delta\Delta G < -0.5$ Stable	Recall
T2837 + TP	-0.00	4804	0.14	0.38	0.48	0.78
T2837 + TP	-0.25	2766	0.11	0.26	0.63	0.64
T2837 + TP	-0.50	1770	0.08	0.18	0.74	0.48
T2837 + TP	-0.75	1182	0.07	0.14	0.80	0.35
T2837 + TP	-1.00	804	0.06	0.10	0.84	0.25
T2837 + TP	-1.50	280	0.04	0.06	0.90	0.13
T2837	-0.00	503	0.27	0.45	0.28	0.51
T2837	-0.25	198	0.23	0.34	0.42	0.30
T2837	-0.50	72	0.24	0.25	0.51	0.13
T2837	-0.75	36	0.33	0.19	0.47	0.06
T2837	-1.00	18	0.22	0.28	0.50	0.03
T2837	-1.50	2	0.00	0.50	0.50	0.00

(a)

DataSet	Predict $\Delta\Delta G$ < Threshold	#Data	Experimental $\Delta\Delta G > 0.5$ Destable	Experimental $\|\Delta\Delta G\| \leq 0.5$ Neutral	Experimental $\Delta\Delta G < -0.5$ Stable	Recall
T2837+TP	0.00	2054	0.17	0.37	0.47	0.75
T2837+TP	-0.25	1677	0.12	0.27	0.60	0.61
T2837+TP	-0.50	1298	0.10	0.19	0.71	0.47
T2837+TP	-0.75	1015	0.07	0.14	0.78	0.37
T2837+TP	-1.00	767	0.06	0.11	0.83	0.28
T2837+TP	-1.50	473	0.04	0.05	0.91	0.17
T2837	0.00	132	0.37	0.42	0.21	0.48
T2837	-0.25	71	0.36	0.33	0.30	0.26
T2837	-0.50	41	0.35	0.25	0.40	0.15
T2837	-0.75	22	0.38	0.18	0.44	0.08
T2837	-1.00	13	0.40	0.17	0.43	0.05
T2837	-1.50	6	0.42	0.08	0.50	0.02

(b)

DataSet	Predict $\Delta\Delta G$ < Threshold	#Data	Experimental $\Delta\Delta G > 0.5$ Destable	Experimental $\|\Delta\Delta G\| \leq 0.5$ Neutral	Experimental $\Delta\Delta G < -0.5$ Stable	Recall
T2837 + TP	-0.00	2540	0.17	0.39	0.44	0.41
T2837 + TP	-0.25	1624	0.14	0.34	0.52	0.31
T2837 + TP	-0.50	928	0.12	0.30	0.58	0.20
T2837 + TP	-0.75	524	0.10	0.25	0.65	0.12
T2837 + TP	-1.00	261	0.10	0.21	0.69	0.07
T2837 + TP	-1.50	64	0.09	0.11	0.80	0.02
T2837	-0.00	422	0.37	0.41	0.22	0.34
T2837	-0.25	216	0.36	0.37	0.28	0.22
T2837	-0.50	96	0.35	0.34	0.30	0.11
T2837	-0.75	44	0.36	0.34	0.30	0.05
T2837	-1.00	19	0.47	0.26	0.26	0.02
T2837	-1.50	5	0.40	0.00	0.60	0.01

(c)

Supplementary Table 8. (a) The experimental distribution of Stability Oracle on T2837 and T2837 + TP with different prediction $\Delta\Delta G$ thresholds. (b) The experimental distribution of Prostata-IFML on T2837 and T2837 + TP with different prediction $\Delta\Delta G$ thresholds. (c) The experimental distribution of RaSP on T2837 and T2837 + TP with different prediction $\Delta\Delta G$ thresholds.

A.10 Regression and classification performance on T2837 and its augmentations

Test Set	#Mutations	*Pearson*	*Spearman*	*RMSE*	*MCC*	*AUROC*	Precision	Recall	Accuracy
T2837 Orig	2837	0.59	0.62	1.65	0.39	0.81	0.55	0.46	0.82
T2837 TR	2830	0.59	0.62	1.65	0.39	0.81	0.86	0.82	0.82
T2837 Orig + TR	5660	0.74	0.78	1.65	0.63	0.90	0.81	0.83	0.82
T2837 TP	7698	0.6556	0.65	1.43	0.47	0.81	0.73	0.74	0.7371
T2837 Orig + TP	10528	0.68	0.68	1.53	0.50	0.84	0.69	0.68	0.77
T2837 Orig + TP + TR	13358	0.70	0.72	1.53	0.54	0.85	0.76	0.78	0.77

(a)

Test Set	#Mutations	Pearson	Spearman	RMSE	MCC	AUROC	Precision	Recall	Accuracy
T2837 Orig	2837	0.53	0.52	1.77	0.31	0.75	0.43	0.47	0.77
T2837 TR	2837	0.53	0.52	1.77	0.30	0.75	0.86	0.83	0.76
T2837 Orig + TR	5674	0.69	0.69	1.77	0.54	0.85	0.77	0.76	0.76
T2837 TP	7720	0.67	0.64	1.40	0.43	0.80	0.72	0.71	0.71
T2837 Orig + TP	10557	0.66	0.64	1.51	0.45	0.81	0.68	0.68	0.73
T2837 Orig + TP + TR	13394	0.68	0.66	1.57	0.48	0.82	0.74	0.73	0.73

(b)

Test Set	#Mutations	*Pearson*	*Spearman*	*RMSE*	*MCC*	*AUROC*	Precision	Recall	Accuracy
T2837 Orig	2830	0.57	0.54	1.56	0.25	0.61	0.44	0.33	0.79
T2837 TR	2830	0.23	0.27	2.40	0.16	0.60	0.86	0.51	0.55
T2837 Orig + TR	5660	0.55	0.54	2.02	0.36	0.67	0.76	0.48	0.67
T2837 TP	7714	0.47	0.41	1.98	0.24	0.61	0.69	0.38	0.61
T2837 Orig + TP	10544	0.49	0.46	1.87	0.27	0.62	0.65	0.38	0.66
T2837 Orig + TP + TR	13374	0.49	0.47	1.20	0.29	0.63	0.72	0.42	0.63

(c)

Supplementary Table 9. (a) Stability Oracle's regression and classification metrics on T2837 and its augmented datasets. TR: Thermodynamic Reversibility augmentation, TP: Thermodynamic Permutation augmentation. Note: mutations from proteins that failed the data engineering pipeline are excluded. (b) Prostata-IFML's regression and classification metrics on T2837 and its augmented datasets. (c) RaSP's regression and classification metrics on T2837 and its augmented datasets. Note: we cannot calculate MCC and AUROC for the experimental stable dataset since there is only a single label. Note: mutations from proteins that failed the data engineering pipeline are excluded.

A.11 The distribution for Stability Oracle's predicted $\Delta\Delta G$ v.s. experimental $\Delta\Delta G$ on T2837 + TP.

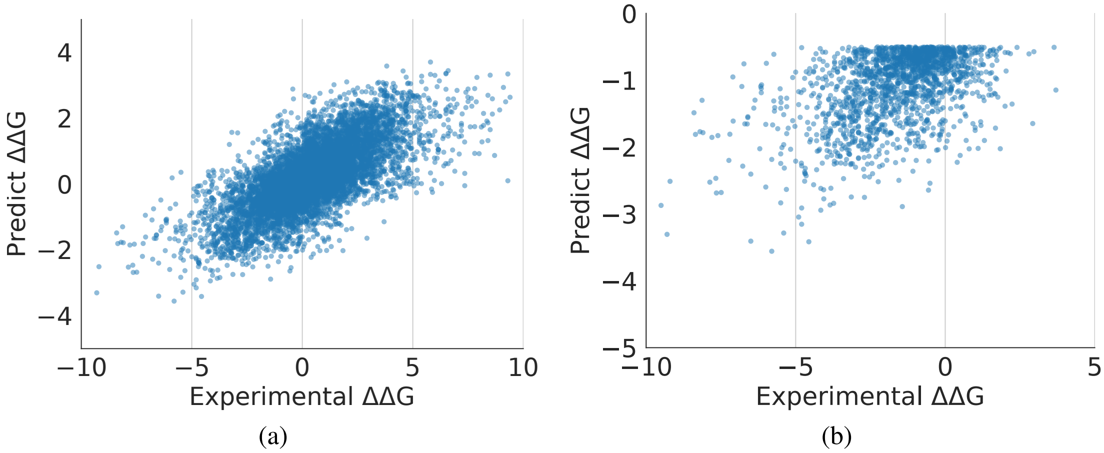

Supplementary Figure 6. (a) shows Stability Oracle's $\Delta\Delta G$ predictions vs experimental $\Delta\Delta G$ measurements on T2837 (RMSE is 1.53 kcal/mol as displayed in Table 9a for T2837 Orig + TP). (b) shows the stable prediction subset of (a), where the $\Delta\Delta G$ prediction is $<$ -0.5 kcal/mol.

A.12 UMAP visualization of Stability Oracle's hidden representation

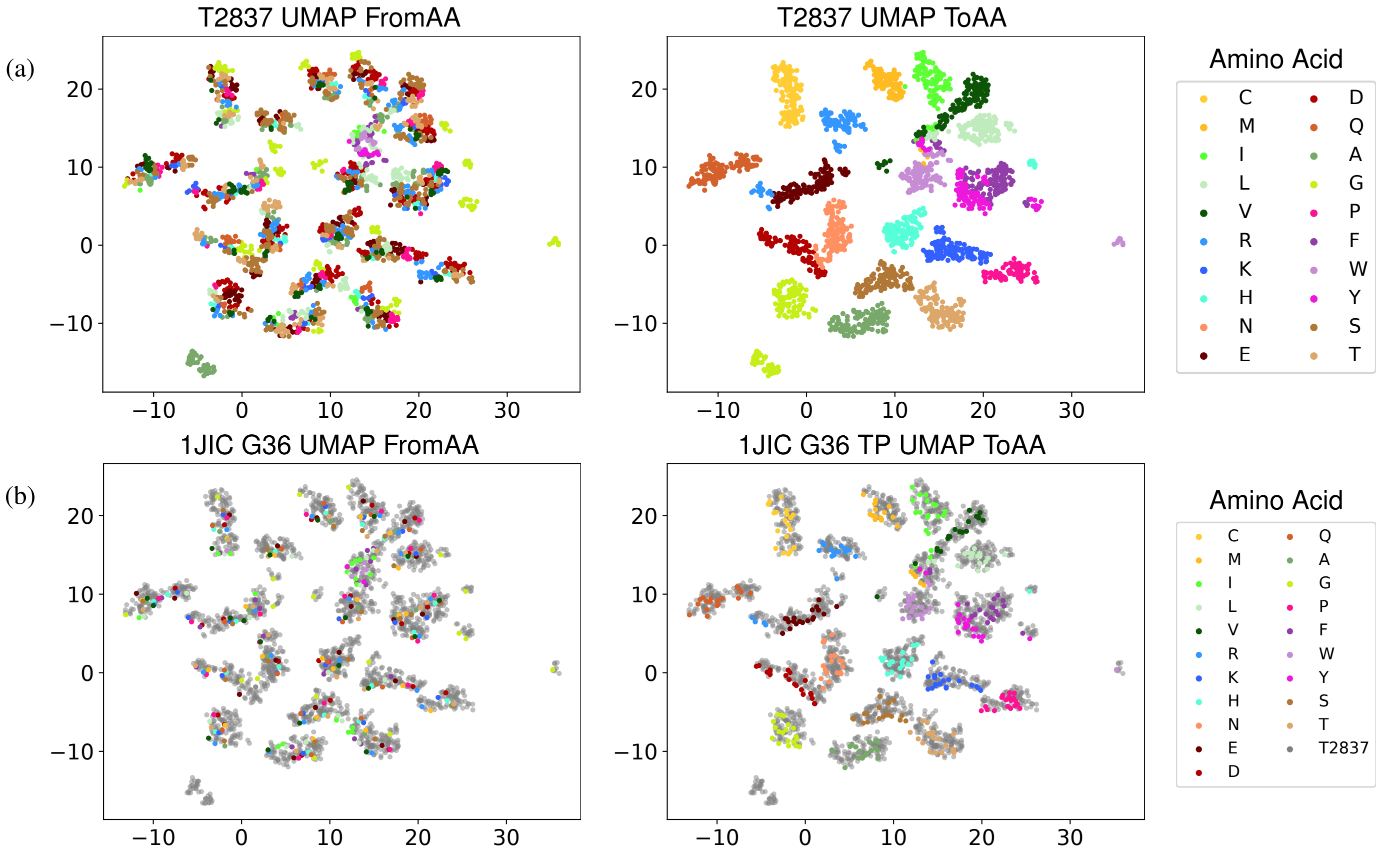

Supplementary Figure 7. UMAP visualization of Stability Oracle's 128-dim mutation hidden representation. a) UMAP of T2837 colored by the "FromAA" (left) and "ToAA" (right). b) UMAP of all 380 mutations types for G36 in protein PDB: 1JIC overlayed with the T2837 as background (gray). In the left and right figures, the points are colored by their "fromAA" and "toAA" amino acid type, respectively.

A.13 Literature Comparisons

Metric	Method	P53 (2OCJ)		Myoglobin (1BZ6)		S-sym		S669	
		Forward	Inverse	Forward	Inverse	Forward	Inverse	Forward	Inverse
Pearson	ThermoMPNN[1]	**0.76**	-	0.60	-	0.72	0.60	0.43	-
	ThermoMPNN*	0.66	0.57	0.58	0.49	0.66	0.58	0.40	0.34
	Stability Oracle	0.73	**0.73**	**0.68**	**0.68**	**0.72**	**0.72**	**0.52**	**0.52**
Spearman	ThermoMPNN[1]	**0.70**	-	0.60	-	-	-	-	-
	ThermoMPNN*	0.61	0.54	0.58	0.50	0.64	0.55	0.41	0.34
	Stability Oracle	0.68	**0.66**	**0.66**	**0.66**	**0.70**	**0.70**	**0.53**	**0.53**
AUROC	ThermoMPNN*	0.76	0.73	0.77	0.74	0.83	0.78	0.72	0.69
	Stability Oracle	**0.80**	**0.80**	**0.82**	**0.81**	**0.87**	**0.87**	**0.75**	**0.75**
RMSE	ThermoMPNN[1]	-	-	-	-	**1.12**	1.53	1.52	-
	ThermoMPNN*	**1.49**	1.78	0.95	1.04	1.17	1.59	1.56	1.84
	Stability Oracle	**1.50**	**1.51**	**0.90**	**0.89**	1.22	**1.19**	**1.43**	**1.43**

Supplementary Table 10. Comparison with ThermoMPNN. ThermoMPNN[1] is the performance reported in the ThermoMPNN preprint and ThermoMPNN* is performance we obtained from the model checkpoint in their official GitHub repository:https://github.com/Kuhlman-Lab/ThermoMPNN/tree/main/models. We did not evaluate the extent of data leakage with these test sets for ThermoMPNN.

Method	P53 (2OCJ)		Myoglobin (1BZ6)		S-sym		S669		T2837	
	Forward	Inverse	Forward	Inverse	Forward	Inverse	Forward	Inverse	Forward	Inverse
Stability Oracle	0.73	0.73	0.68	0.68	0.72	0.72	0.52	0.52	0.59	0.59
PROSTATA-IFML	0.73	0.73	0.55	0.55	0.56	0.56	0.51	0.51	0.53	0.53
ThermoMPNN[2]	0.66	0.57	0.58	0.49	0.66	0.58	0.40	0.34	-	-
ThermoNet[3]	0.45	0.56	0.38	0.37	0.47	0.47	0.39	0.38	-	-
ProsGNN[4]	-	-	0.48	0.43	0.61	0.56	-	-	-	-
DDGUN[5]	-	-	-	-	0.48	0.48	0.41	0.38	-	-
Premps[6]	-	-	-	-	-	-	0.41	0.42	-	-
Rosetta[7]	-	-	-	-	0.68	0.43	0.39	0.40	-	-
DynaMut[8]	-	-	-	-	-	-	0.41	0.34	-	-
SDM[9]	-	-	-	-	-	-	0.41	0.13	-	-
FoldX[10]	-	-	-	-	0.63	0.39	-	-	-	-
RaSP[11]	0.67	0.10	0.68	0.36	0.64	0.30	0.39	0.27	0.55	0.23
DUET[12]	-	-	-	-	0.63	0.13	0.41	0.23	-	-

Supplementary Table 11. Pearson correlation coefficient (PCC) comparison of Stability Oracle and Prostata-IFML against literature computational stability predictors.

A Overview of Deep Learning for Stability Prediction

The lack of available stability data has prevented deep learning frameworks from significantly accelerating the field and the community still primarily relies on physics-based methods, such as Rosetta[7] and FoldX[10], or shallow machine learning approaches[9,12–19]. Nonetheless, several deep learning frameworks for stability predictions have been published and they can be broadly categorized by their input type: sequence or structure. The current structure frameworks include DeepDDG[20], ThermoNet[3], ProSGNN[4] , and AC-DNN[21] and sequence frameworks include Prostata[22] and HotProteins[23]. While both structure and sequence frameworks are competitive with current state-of-the-art methods, the two types have their own benefits and limitations. The structure frameworks capture atomic interactions within a protein and with other biomolecules, such as ligands, nucleotides, and other proteins. Furthermore, the richness of atomic representations enables generalization while training with only 10^3-10^4 protein structures. However, all existing frameworks require an additional mutant structure to make a stability prediction. This adds a significant computational hurdle that scales linearly with the number of inferences. For example, to conduct a computational deep mutational scan (DMS) of a 300 amino acid protein, one must computationally generate 5700 mutant structures via AlphaFold2[24] or Rosetta[7] being the most prevalent tools used in the literature. For sequence-based frameworks their primary benefit is that they required little data engineering and have billions of primary sequences readily available for training. This gives them a low barrier to entry and makes them popular amongst the machine learning community, resulting in an explosion of transformer-based large language models (LLMs) across the protein community[22,25–33]. There are two primary limitations to sequence models. First, LLMs require extensive training on large sequence databases (UniRef50) to learn meaningful representations for downstream applications. Training these models is very computational expensive and only affordable to well-funded academics and technology companies[25–27,29,31,33]. Second, the self-supervised pre-training of transformer models constrains the training space to single protein sequences. As protein engineers, we intuitively understand that proteins are more than their primary sequence; proteins often form complexes, undergo post-translational modifications, and interact with cofactors, DNA, RNA, and oligosaccharides. This artificial domain shift limits their generalization in downstream task and has already been empirically demonstrated when predicting stability changes at protein-protein and protein-ligand interfaces[22].

B Data and Metric issues in Computational Stability Prediction

B.1 Data

Systematic analysis of computational stability predictors published over the last 15 years has identified several important shortcomings hindering the field from taking advantage of deep learning algorithms. These shortcomings are present in every facet of the data pipeline: data scarcity, data bias, data leakage, poor metrics for evaluating performance, and computational cost. Here is a high-level, non-exhaustive overview of the most pressing issues. The limited amount of experimental data is heavily biased in several ways: 1) dominated by destabilizing mutations, resulting in only ∼20% of point mutations predicted to stabilize indeed increase stability[34–36]; 2) stabilizing mutations are narrowly concentrated between -2 to 0 kcal/mol, causing models to overfit to this distribution and underestimate and not generalize to strongly stabilizing mutations[34–36]; 3) stabilizing mutations are enriched with surface hydrophobic mutations, causing stabilizing predictions to often unintentionally lower protein solubility[34–36] 4) of the 380 types of amino acid substitutions ∼20-70% have no data in the common training and validation sets and the ones sampled are significantly biased toward mutations "from" or "to" alanine and valine[35]. Due to the hierarchical nature of proteins, splitting experimental data into training and validation sets can be done at the mutation, position, protein, and sequence cluster levels. All but sequence cluster result in data leakage, thus, most published computational tools report inflated evaluation metrics and display poor generalization.

B.2 Metrics

The common metrics used to assess the performance of computational stability predictors (Pearson correlation, classification accuracy, and error) are not appropriate for the goal of accurately identifying stabilizing mutations and tend to be misleading[34–36]. The metrics naively formulate the problem as a regression or classification task and are not tailored to the actual goal: accurately predicting stabilizing mutations. This misalignment has resulted in metrics that do not prioritize improvement in accurately predicting stabilizing mutations and their game-ability constantly results in inflated reported performance metrics that fail to generalize upon evaluation by third-party researchers. This game-ability and in turn poor generalization for stabilizing mutations is demonstrated in great detail in a study that consisted of 21 established computational tools[35]. Here, the authors demonstrate how Spearman and Matthew correlation (MCC) can correctly identify deficiencies in an overly simplified model while the original metrics couldn't. The overly simplified model used was an artificial neural network with a single, two-neuron hidden layer that takes four commonly used structural descriptors of amino acid substitution as input: change in volume, change in hydrophobicity, change in secondary structure propensity, and location via solvent exposure. For a more in-depth metric comparison, we refer the reader to[35]. We argue that these metrics are still insufficient for monitoring stabilizing mutation predictive improvement. Here, we use classification metrics, such as precision, recall, and AUROC to monitor improvements for identifying stabilizing mutations during the development of Stability Oracle.

References

1. Nisthal, A., Wang, C. Y., Ary, M. L. & Mayo, S. L. Protein stability engineering insights revealed by domain-wide comprehensive mutagenesis. *Proc. Natl. Acad. Sci.* **116**, 16367–16377 (2019).
2. Dieckhaus, H., Brocidiacono, M., Randolph, N. & Kuhlman, B. Transfer learning to leverage larger datasets for improved prediction of protein stability changes. *bioRxiv* (2023).
3. Li, B., Yang, Y. T., Capra, J. A. & Gerstein, M. B. Predicting changes in protein thermodynamic stability upon point mutation with deep 3d convolutional neural networks. *PLoS computational biology* **16**, e1008291 (2020).
4. Wang, S., Tang, H., Shan, P. & Zuo, L. Pros-gnn: Predicting effects of mutations on protein stability using graph neural networks. *bioRxiv* 2021–10 (2021).
5. Montanucci, L., Capriotti, E., Frank, Y., Ben-Tal, N. & Fariselli, P. Ddgun: an untrained method for the prediction of protein stability changes upon single and multiple point variations. *BMC bioinformatics* **20**, 1–10 (2019).
6. Chen, Y. *et al.* Premps: Predicting the impact of missense mutations on protein stability. *PLoS computational biology* **16**, e1008543 (2020).
7. Kellogg, E. H., Leaver-Fay, A. & Baker, D. Role of conformational sampling in computing mutation-induced changes in protein structure and stability. *Proteins: Struct. Funct. Bioinforma.* **79**, 830–838 (2011).
8. Rodrigues, C. H., Pires, D. E. & Ascher, D. B. Dynamut2: Assessing changes in stability and flexibility upon single and multiple point missense mutations. *Protein Sci.* **30**, 60–69 (2021).
9. Worth, C. L., Preissner, R. & Blundell, T. L. Sdm—a server for predicting effects of mutations on protein stability and malfunction. *Nucleic acids research* **39**, W215–W222 (2011).

10. Schymkowitz, J. *et al.* The foldx web server: an online force field. *Nucleic acids research* **33**, W382–W388 (2005).

11. Blaabjerg, L. M. *et al.* Rapid protein stability prediction using deep learning representations. *eLife* **12**, e82593 (2023).

12. Pires, D. E., Ascher, D. B. & Blundell, T. L. Duet: a server for predicting effects of mutations on protein stability using an integrated computational approach. *Nucleic acids research* **42**, W314–W319 (2014).

13. Capriotti, E., Fariselli, P. & Casadio, R. I-mutant2. 0: predicting stability changes upon mutation from the protein sequence or structure. *Nucleic acids research* **33**, W306–W310 (2005).

14. Pires, D. E., Ascher, D. B. & Blundell, T. L. mcsm: predicting the effects of mutations in proteins using graph-based signatures. *Bioinformatics* **30**, 335–342 (2014).

15. Laimer, J., Hofer, H., Fritz, M., Wegenkittl, S. & Lackner, P. Maestro-multi agent stability prediction upon point mutations. *BMC bioinformatics* **16**, 1–13 (2015).

16. Dehouck, Y., Kwasigroch, J. M., Gilis, D. & Rooman, M. Popmusic 2.1: a web server for the estimation of protein stability changes upon mutation and sequence optimality. *BMC bioinformatics* **12**, 1–12 (2011).

17. Wainreb, G., Wolf, L., Ashkenazy, H., Dehouck, Y. & Ben-Tal, N. Protein stability: a single recorded mutation aids in predicting the effects of other mutations in the same amino acid site. *Bioinformatics* **27**, 3286–3292 (2011).

18. Romero, P. A., Krause, A. & Arnold, F. H. Navigating the protein fitness landscape with gaussian processes. *Proc. Natl. Acad. Sci. USA* **110**, E193–E201 (2013).

19. Cheng, J., Randall, A. & Baldi, P. Prediction of protein stability changes for single-site mutations using support vector machines. *Proteins: Struct. Funct. Bioinforma.* **62**, 1125–1132 (2006).

20. Cao, H., Wang, J., He, L., Qi, Y. & Zhang, J. Z. Deepddg: Predicting the stability change of protein point mutations using neural networks. *J. Chem. Inf. Model.* **59**, 1508–1514 (2019).

21. Benevenuta, S., Pancotti, C., Fariselli, P., Birolo, G. & Sanavia, T. An antisymmetric neural network to predict free energy changes in protein variants. *J. Phys. D: Appl. Phys.* **54**, 245403 (2021).

22. Umerenkov, D. *et al.* PROSTATA: a framework for protein stability assessment using transformers. *Bioinformatics* **39**, btad671, DOI: 10.1093/bioinformatics/btad671 (2023).

23. Chen, T. *et al.* Hotprotein: A novel framework for protein thermostability prediction and editing. In *The Eleventh International Conference on Learning Representations* (OpenReview.net, 2023).

24. Jumper, J. *et al.* Highly accurate protein structure prediction with alphafold. *Nature* **596**, 583–589 (2021).

25. Rives, A. *et al.* Biological structure and function emerge from scaling unsupervised learning to 250 million protein sequences. *PNAS* DOI: 10.1101/622803 (2019).

26. Meier, J. *et al.* Language models enable zero-shot prediction of the effects of mutations on protein function. *Adv. Neural Inf. Process. Syst.* **34**, 29287–29303 (2021).

27. Lin, Z. *et al.* Evolutionary-scale prediction of atomic-level protein structure with a language model. *Science* **379**, 1123–1130, DOI: 10.1126/science.ade2574 (2023).

28. Nijkamp, E., Ruffolo, J. A., Weinstein, E. N., Naik, N. & Madani, A. Progen2: exploring the boundaries of protein language models. *Cell systems* **14**, 968–978 (2023).

29. Notin, P. *et al.* Tranception: protein fitness prediction with autoregressive transformers and inference-time retrieval. In *International Conference on Machine Learning*, 16990–17017 (PMLR, 2022).

30. Rao, R. M. *et al.* Msa transformer. In *International Conference on Machine Learning*, 8844–8856 (PMLR, 2021).

31. Notin, P. M. *et al.* Trancepteve: Combining family-specific and family-agnostic models of protein sequences for improved fitness prediction. *bioRxiv* 2022–12 (2022).

32. Hie, B. L. *et al.* Efficient evolution of human antibodies from general protein language models. *Nat. Biotechnol.* **42**, 275–283 (2024).

33. Elnaggar, A. *et al.* Prottrans: Toward understanding the language of life through self-supervised learning. *IEEE transactions on pattern analysis machine intelligence* **44**, 7112–7127 (2021).

34. Pucci, F., Schwersensky, M. & Rooman, M. Artificial intelligence challenges for predicting the impact of mutations on protein stability. *Curr. opinion structural biology* **72**, 161–168 (2022).

35. Broom, A., Trainor, K., Jacobi, Z. & Meiering, E. M. Computational Modeling of Protein Stability: Quantitative Analysis Reveals Solutions to Pervasive Problems. *Structure* **28**, 717–726.e3, DOI: 10.1016/j.str.2020.04.003 (2020).

36. Benevenuta, S., Birolo, G., Sanavia, T., Capriotti, E. & Fariselli, P. Challenges in predicting stabilizing variations: An exploration. *Front. Mol. Biosci.* **9**, 1–10, DOI: 10.3389/fmolb.2022.1075570 (2023).
